# Symptom burden survey and symptom clusters in patients with cervical cancer: a cross-sectional survey

**DOI:** 10.1007/s00520-023-07802-7

**Published:** 2023-05-16

**Authors:** Kai-nan Zhou, Yan Wang, Yi Xie, Shu-han Yang, Su-ying Liu, Yu-hang Fang, Ying Zhang

**Affiliations:** 1 Guang’anmen Hospital, China Academy of Chinese Medical Sciences, No. 5, Beixian Ge Street, Xicheng District, Beijing, 100053 China; 2 Graduate School, Beijing University of Chinese Medicine, No. 11, Beisanhuan Dong Road, Chaoyang District, Beijing, 100029 China

**Keywords:** Symptom burden, Symptom clusters, Cervical cancer, Radiotherapy, Chemotherapy, Exploratory factor analysis

## Abstract

**Purpose:**

The purpose of this study is to determine the incidence and severity of symptoms of patients with cervical cancer within 6 months after radiotherapy and chemotherapy, form a symptom burden report, evaluate the distribution characteristics of symptoms, identify symptom clusters, and provide a basis for clinical doctors and nurses to improve the symptom management of patients with cervical cancer after radiotherapy and chemotherapy.

**Methods:**

The patients with cervical cancer within 6 months after radiotherapy and chemotherapy were recruited to investigate their symptom burden. Exploratory factor analysis was used to identify symptom clusters.

**Results:**

A total of 250 patients participated in the study. The study found that the most common symptom among the 40 symptoms was fatigue, and the most serious symptom was nocturia. Based on the occurrence rate and severity of symptoms, nine symptom clusters were identified, including psycho–emotion-related symptom cluster, pain–disturbed sleep-related symptom cluster, menopausal symptom cluster, tinnitus–dizziness-related symptom cluster, urinary-related symptom cluster, dry mouth–bitter taste-related symptom cluster, intestinal-related symptom cluster, memory loss–numbness-related symptom cluster, and emaciation-related symptom cluster. The three most serious symptom clusters are pain–disturbed sleep-related symptom cluster, urinary-related symptom cluster, and memory loss–numbness-related symptom cluster.

**Conclusion:**

The symptoms of patients with cervical cancer within 6 months after radiotherapy and chemotherapy are complex, and nine symptom clusters can be identified according to the incidence and severity of symptoms. We can find the potential biological mechanism of each symptom cluster through the discussion of previous mechanism research and clinical research. The number of symptom clusters and the number of symptoms within the symptom cluster are closely related to the symptom evaluation scale selected for the study. Therefore, the symptom cluster study urgently needs a targeted symptom evaluation scale that can comprehensively reflect the patient’s condition.

## Introduction

Cervical cancer has a high incidence rate and mortality, which seriously threatens women’s health and life. The 2020 global cancer statistics report points out that the incidence rate of cervical cancer among women in the world is 6.5%, and the mortality rate is 7.7%, both ranking fourth in female cancer [1]. Cervical cancer is insidious and has no typical symptoms at the early stage. Two-thirds of cervical cancer patients have developed to local middle and late stage at the time of initial diagnosis. The treatment method at this stage is mainly synchronous radiotherapy and chemotherapy [2]. Cervical cancer patients receiving radiotherapy and chemotherapy may have symptoms of multiple organs and systems with different severity and distress, which seriously affect the physical and mental health and quality of life of patients, and even cause patients to stop treatment. Symptom cluster, as a new multi-dimensional symptom management model, has gradually become a research hotspot in the field of cancer. First, it can provide medical staff with evidence for symptom management to prolong the survival period of patients and improve their quality of life; second, it can provide evidence for finding common mechanisms between symptoms [3]; third, it can evaluate the effect of certain treatment methods [4]. Symptom cluster is composed of two or more interrelated symptoms [5, 6]. The symptoms in the symptom cluster are characterized by compactness, simultaneity, and stability [7] and may have a common biological mechanism [8]. Research on cancer symptom cluster has made progress in symptom cluster identification and subgroup analysis [9, 10], stability analysis of symptom cluster [11], discussion on biological mechanism of symptom cluster, and clinical intervention of symptom cluster [12, 13].

At present, there is little research on the symptom cluster of cervical cancer patients. In one study, 286 cervical cancer patients receiving concurrent radiotherapy and chemotherapy were investigated with Anderson Symptom Inventory, Brief Illness Perception Questionnaire (BIPQ) and Functional Assessment Cancer Therapy-Cervix (FACT Cx), and four symptom clusters were identified, including psychological status symptom cluster, therapy side-effect symptom cluster, sickness symptom cluster, and gastrointestinal symptom cluster [14]. Another study used the Chinese version of the 13-item M.D. Anderson Symptom Inventory (MDASI) to analyze the symptom clusters of Chinese cervical cancer patients undergoing radiotherapy, chemoradiotherapy, or postoperative chemoradiotherapy and extracted gastrointestinal symptom cluster, mood-cognitive symptom cluster, sickness-behavior symptom cluster, and pain-related symptom cluster [15]. It is clinically found that the symptom items involved in the scale used in the above studies cannot cover the burden of patients’ symptoms, which may lead to the lack of representativeness and pertinence of the identified symptom clusters. The symptoms caused by radiotherapy and chemotherapy have a certain degree of delay, so how to manage the symptoms of cervical cancer patients after radiotherapy and chemotherapy deserves further attention and research.

This study will collect the symptoms of patients with cervical cancer within 6 months after radiotherapy and chemotherapy as much as possible to form a symptom burden report of patients and further evaluate the distribution characteristics of their symptoms by investigating the two dimensions of symptom incidence and severity, so as to determine their symptom clusters, providing a basis for clinical doctors and nurses to improve the symptom management of patients with cervical cancer after radiotherapy and chemotherapy.

## Methods

### Patients and settings

This is a cross-sectional study to investigate the burden of symptoms of cervical cancer patients within 6 months after receiving radiotherapy and chemotherapy and determine their symptom clusters. The patient was from Guang’anmen Hospital, Chinese Academy of Chinese Medical Sciences. The inclusion criteria of participants were as follows: ① Patients with cervical cancer diagnosed by histopathology; ② ≤ 6 months after completion of radiotherapy and chemotherapy; ③ age ≥ 18 years; ④ informed consent and sign the informed consent form; ⑤ can carry out normal language communication and exchange, and can cooperate with researchers to complete the questionnaire. The exclusion criteria were as follows: ① those with mental or cognitive dysfunction; ② poor tumor control or distant metastasis; ③ complications of other organs were serious and uncontrollable. The exclusion criteria were as follows: ① patients with mental or cognitive dysfunction; ② patients with poor tumor control or distant metastasis; ③ complications were severe and uncontrolled. The patients were recruited from September 2020 to January 2022 in the outpatient or inpatient department of the oncology department of Guang’anmen Hospital. The burden of symptoms was determined by prestudy. The sample size is estimated according to the maximum number of items of the scale used, which should be 5–10 times of the number of items. The clinical symptom questionnaire in this study involves 40 symptom items, so the sample size is at least 200 cases.

### Instruments

The patient completed the demographic questionnaire and disease characteristics questionnaire. The demographic questionnaire includes name, age, contact information, height, weight, nationality, native place, education level, marital status, residence, annual family income, payment method, and treatment cost burden. The disease characteristics questionnaire included pathological type, tumor stage, end time of radiotherapy and chemotherapy, treatment measures received, and concomitant diseases.

The symptom assessment scale is the main tool for cancer symptom cluster identification. In order to comprehensively investigate the symptoms of patients and identify their symptom clusters, this study formed a clinical symptom questionnaire by investigating the symptom burden of 40 patients with cervical cancer within 6 months after radiotherapy and chemotherapy. The evaluation period of the survey is the patient’s symptom burden in the past week. The specific process is as follows: (1) investigate the symptoms of 30 patients, extract the symptoms with a symptom incidence rate ≥ 5%, and initially form a clinical symptom questionnaire; (2) another 10 patients were observed. If new symptoms with high frequency were found, they should be added. The severity of symptoms in the clinical symptom questionnaire is measured as none, mild, moderate, and severe according to the patient’s self-feelings, and 0 means “none,” 1 means “mild,” 2 means “moderate,” and 3 means “severe.” The total score range is 0 to 3 points. The average score calculation method for each item is to add up the scores of all patients in the item and then divide by the number of patients.

### Study procedures

The study obtained the ethical approval of Guang’anmen Hospital, Chinese Academy of Chinese Medical Sciences (2021-116-KY). The research process followed the Helsinki Declaration of the World Medical Congress and other relevant provisions. Before each surveyed person is selected for this study, the researcher is responsible for fully and comprehensively introducing the purpose, procedure, and possible risks of this study to the subjects or their representatives, and to let the surveyed persons know that they have the right to withdraw from this study. After being informed, the subjects or their representatives signed a written informed consent. Demographic questionnaire and disease characteristics questionnaire are completed by the subject or his agent or obtained through electronic medical record. The patient completed the clinical symptom questionnaire at the time of treatment or after admission with the help of the investigator. After the patient completes the questionnaire, the researcher ensures that the questionnaire is completed. The patient’s personal privacy and data confidentiality will be protected during the study.

### Data analysis

SPSS24.0 statistical software was used for data analysis, which was entered into the computer by two people and checked. Cases with missing data were excluded. The internal consistency coefficient for the clinical symptom questionnaires was determined by calculating Cronbach’s alpha. Descriptive statistics and frequency distribution calculation were carried out for the demographic questionnaire and disease characteristics questionnaire. Symptom cluster recognition methods mainly include principal component analysis (PCA), confirmative factor analysis (CFA), hierarchical cluster analysis (HCA), and exploratory factor analysis (EFA). In this study, EFA was used to identify symptom clusters for 40 symptom items in the clinical symptom questionnaire. The mean and standard deviation were used to get the severity of each symptom cluster. The incidence and severity of symptoms were analyzed by means of mean, standard deviation, and constituent ratio. Hypothesis tests were conducted on both sides, with *P* < 0.05 as the statistical difference.

In order to improve the specificity and clinical significance of the symptom clusters identified based on the clinical symptom questionnaire, and to make enough variation and covariates in the data, the study excluded symptoms with an incidence rate of less than 30%, and the load of each symptom should be ≥ 0.40. KMO test and Bartlett’s spherical test were performed on the data to determine whether it was suitable for factor analysis. The principal component analysis was used to determine the factors. The components whose eigenvalues were greater than 1 were taken as the principal components, and the factors were rotated by the maximum variance method. Finally, symptom clusters were extracted and identified according to the severity of each symptom.

## Results

### Prestudy: clinical questionnaire based on patients’ symptom burden

#### Demographic and clinical characteristics

In the prestudy of the symptom burden survey of 30 patients, 21 (70%) were aged 40 to 60 years, 12 (40%) had an education level of junior high school, and 28 (93.3%) were married. Most patients had squamous cell carcinoma (76.7%), tumor stage II (33.3%), stage III (43.3%), had received radiotherapy and chemotherapy (56.7%), and had no comorbid other diseases (83.3%) (Table 1). No demographic changes were evident in the prestudy of the additional 10 patient symptom burden survey (Table 2).

**Table 1:** Demographic and clinical characteristics (*n* = 30)

Characteristic		*n*	%
Age in years	30⁓39	3	10.0
	40⁓49	10	33.3
	50⁓59	11	36.7
	60⁓69	5	16.7
	70⁓79	1	3.3
Height (cm), mean (SD)		154.38(29.15)	
Weight (kg), mean (SD)		58.53(8.53)	
Degree of education	Junior high school	12	40.0
	University or above	3	10.0
	junior college	5	16.7
	High school/technical secondary school	9	30.0
	Primary school and below	1	3.3
Marital status	Married	28	93.3
	Divorce	2	6.7
Place of residence	City	18	60.0
	County district	6	20.0
	Countryside	6	20.0
Level of treatment cost burden	No	5	16.7
	Moderate	17	56.7
	Severe	8	26.7
Pathological type	Squamous cell carcinoma	23	76.7
	Adenocarcinoma	5	16.7
	Adeno-squamous carcinoma	2	6.7
Tumor stage	I	7	23.3
	II	10	33.3
	III	13	43.3
	IV	0	0
Treatment already received	Radiotherapy and chemotherapy	17	56.7
	Surgery, radiotherapy, and chemotherapy	13	43.3
Whether comorbid chronic diseases	No	25	83.3
	Hypertension	1	3.3
	Diabetes	2	6.7
	Other	2	6.7

*SD* standard deviation

**Table 2:** Demographic and clinical characteristics (*n* = 10)

Characteristic		*n*	%
Age in years	30⁓39	1	10.0
	40⁓49	3	30.0
	50⁓59	4	40.0
	60⁓69	2	20.0
Height (cm), mean (SD)		162.50 (4.38)	
Weight (kg), mean (SD)		60.10 (4.61)	
Degree of education	Junior high school	4	40.0
	University or above	0	0
	junior college	1	10.0
	High school/technical secondary school	4	40.0
	Primary school and below	1	3.3
Marital status	Married	9	90.0
	Divorce	1	10.0
Place of residence	City	6	60.0
	County district	3	30.0
	Countryside	1	10.0
Level of treatment cost burden	No	3	30.0
	Moderate	6	60.0
	Severe	1	10.0
Pathological type	Squamous cell carcinoma	8	80.0
	Adenocarcinoma	2	20.0
	Adeno-squamous carcinoma	0	0
Tumor stage	I	2	20.0
	II	4	40.0
	III	4	40.0
	IV	0	0
Treatment already received	Radiotherapy and chemotherapy	6	60.0
	Surgery, radiotherapy, and chemotherapy	4	40.0
Whether comorbid chronic diseases	No	9	90.0
	Hypertension	1	10.0

*SD* standard deviation

#### Symptom incidence

In the prestudy of the symptom burden survey of 30 patients, 42 symptoms were reported with an incidence of 86.7⁓3.3%, of which the five most common symptoms were memory loss, nocturia, fatigue, distress, and dry mouth, and the five least occurring symptoms were edema, irregular menstruation, bloody purulent stool, fever, and halitosis. After the additional investigation of 10 patients, the prestudy of the symptom burden investigation of 40 patients showed that one new symptom was skin itching, and the five most common symptoms were fatigue, memory loss, restless sleep, nocturia, and dry mouth. The symptoms with an incidence of less than 5% were fever, skin itching, and halitosis (Tables 3 and 4).

**Table 3:** Symptom incidence (*n* = 30)

Symptom	*n* (%)	Symptom	*n* (%)	Symptom	*n* (%)
Memory loss	26 (86.70)	Drowsiness	15 (50.00)	Hot flushes	9 (30.00)
Fatigue	24 (80.00)	Numbness	15 (50.00)	Urinary urgency	8 (26.70)
Nocturia	23 (76.70)	Chills	14 (46.70)	Urinary pain	8 (26.70)
Distress	23 (76.70)	Abdominal distension	14 (46.70)	Dry stool	8 (26.70)
Dry mouth	23 (76.70)	Night sweats	13 (43.30)	Tinnitus	8 (26.70)
Disturbed sleep	23 (76.70)	Pain	13 (43.30)	Difficult defecation	7 (23.30)
Sadness	22 (76.30)	Vomiting	13 (43.30)	Tenesmus	6 (20.00)
Poor appetite	21 (70.00)	Lower abdominal pain	12 (40.00)	Frequent micturition	5 (16.70)
Spontaneous perspiration	19 (63.30)	loose stool	12 (40.00)	Vaginal bleeding	4 (13.30)
Bitter taste	19 (63.30)	Dizziness	12 (40.00)	Edema	3 (10.00)
Shortness of breath	18 (60.00)	Nausea	12 (40.00)	Irregular menstruation	2 (6.70)
Susceptible sigh	16 (53.30)	chest tightness	11 (36.70)	Bloody purulent stool	2 (6.70)
Lumbosacral soreness	15 (50.00)	Abnormal leucorrhea	11 (36.70)	Fever	1 (3.30)
Restlessness	15 (50.00)	Emaciation	9 (30.00)	Halitosis	1 (3.30)

Based on the results of the symptom burden survey for 40 patients, a clinical symptom questionnaire consisting of 40 symptom entries was finally developed, including pain, fatigue, nausea, disturbed sleep, distress, shortness of breath, memory loss, poor appetite, drowsiness, dry mouth, sadness, vomiting, numbness, lumbosacral soreness, lower abdominal pain, abnormal leucorrhea, vaginal bleeding, irregular menstruation, frequent micturition, urinary urgency, urinary pain, nocturia, difficult defecation, dry stool, loose stool, bloody purulent stool, tenesmus, emaciation, edema, chills, hot flushes, night sweats, spontaneous perspiration, dizziness, tinnitus, restlessness, susceptible sigh, chest tightness, abdominal distension, and bitter taste (Table 4).

**Table 4:** Symptom incidence (*n* = 40)

Symptom	*n* (%)	Symptom	*n* (%)	Symptom	*n* (%)
Fatigue	35 (87.50)	Chills	20 (50.00)	Emaciation	11 (27.50)
Memory loss	35 (87.50)	Drowsiness	19 (47.50)	Hot flushes	11 (27.50)
Disturbed sleep	34 (85.00)	Numbness	19 (47.50)	Tinnitus	10 (25.00)
Nocturia	34 (85.00)	Lower abdominal pain	19 (47.50)	Difficult defecation	9 (22.50)
Dry mouth	31 (77.50)	Loose stool	19 (47.50)	Dry stool	8 (20.00)
Distress	30 (75.00)	Abdominal distension	19 (47.50)	Tenesmus	8 (20.00)
Poor appetite	30 (75.00)	Vomiting	16 (40.00)	Vaginal bleeding	4 (10.00)
Shortness of breath	27 (65.50)	Dizziness	16 (40.00)	Bloody purulent stool	4 (10.00)
Sadness	26 (65.00)	Chest tightness	16 (40.00)	Edema	4 (10.00)
Spontaneous perspiration	24 (60.00)	Nausea	15 (37.50)	Irregular menstruation	3 (7.50)
Bitter taste	24 (60.00)	Night sweats	15 (37.50)	Fever	1 (2.50)
Pain	23 (57.50)	Abnormal leucorrhea	14 (35.00)	Halitosis	1 (2.50)
Lumbosacral soreness	23 (57.50)	Urinary urgency	13 (32.50)	Pruritus	1 (2.50)
Restlessness	23 (57.50)	Frequent micturition	11 (27.50)	—	—
Susceptible sigh	21 (52.50)	Urinary pain	11 (27.50)	—	—

### A survey of patient symptom burden in the first 6 months after chemoradiotherapy for 250 cases of cervical cancer

#### Demographic and clinical characteristics

In the 250 patient symptom burden survey, 154 (61.6%) were aged 40 to 59 years, 76 (30.4%) had an education level of high/middle school, 219 (87.6%) were married, and 150 (60.0%) had a moderate level of treatment cost burden. Most of the patients had squamous cell carcinoma (89.2%), the tumor stage was stage III (42.4%), had received radiotherapy and chemotherapy (60.0%), had no comorbid other diseases (79.2%), and the most comorbid diseases were diabetes (10.8%) and hypertension (9.2%) (Tables 5).

**Table 5:** Demographic and clinical characteristics (*n* = 250)

Characteristic		*n*	%
Age in years	20⁓39	46	18.4
	40⁓59	154	61.6
	60⁓79	50	20.0
Height (cm), mean (SD)		160.23(11.13)	
Weight (kg), mean (SD)		60.54(9.70)	
Degree of education	Junior high school	74	29.6
	University or above	42	16.8
	junior college	39	15.6
	High school/technical secondary school	76	30.4
	Primary school and below	19	7.6
Marital status	Married	219	87.6
	Single	13	5.2
	Divorce	11	4.4
	Widow	7	2.8
Place of residence	City	150	60.0
	County district	60	24.0
	Countryside	40	16.0
Level of treatment cost burden	No	54	21.6
	Moderate	150	60.0
	Severe	46	18.4
Pathological type	Squamous cell carcinoma	223	89.2
	Adenocarcinoma	21	8.4
	Adeno-squamous carcinoma	6	2.4
Tumor stage	I	43	17.2
	II	86	34.4
	III	106	42.4
	IV	15	6.0
Treatment already received	Radiotherapy and chemotherapy	150	60.0
	Surgery, radiotherapy, and chemotherapy	100	40.0
Whether comorbid chronic diseases	No	198	79.2
	Hypertension	27	10.8
	Diabetes	23	9.2
	Other	14	5.6

*SD* standard deviation

#### Symptom burden investigation report

**Fig. 1:**
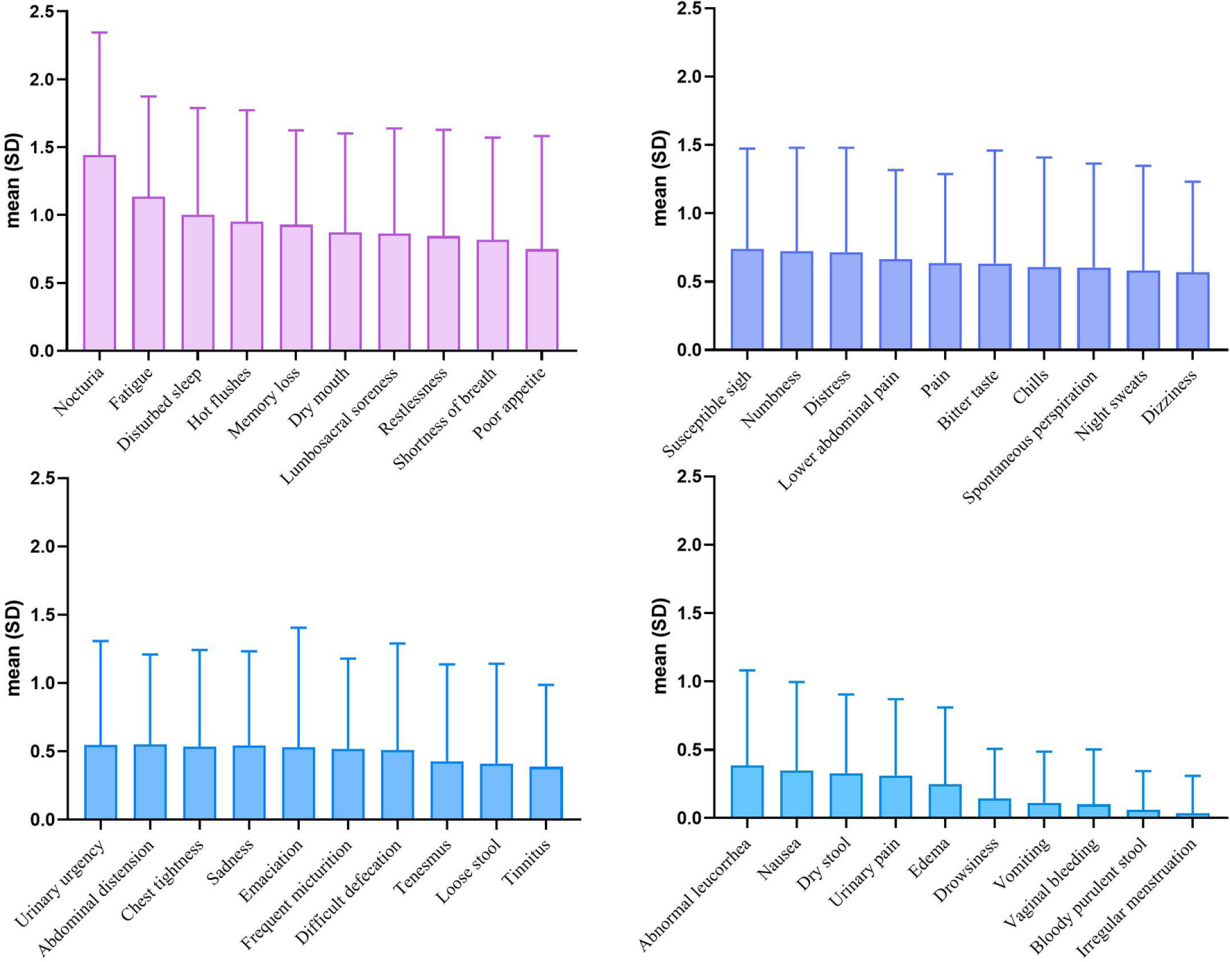
Symptom severity of cervical cancer after radiotherapy and chemotherapy

A calculation of the internal consistency coefficient was performed based on the symptom burden reports formed by the 250 patients, and the internal consistency coefficient is 0.893, this work indicating the questionnaire had good reliability. Of the 40 symptoms reported by the 250 patients, the 5 most common symptoms were fatigue (84.0%), nocturia (83.2%), memory loss (74.4%), disturbed sleep (71.2%), and dry mouth (68.0%), and the 5 most severe symptoms were nocturia (1.44 ± 0.90), fatigue (1.14 ± 0.74), disturbed sleep (1.00 ± 0.78), hot flushes (0.95 ± 0.82), and memory loss (0.93 ± 0.70) (Table 6) (Fig. 1).

**Table 6:** Symptom incidence and severity of cervical cancer after radiotherapy and chemotherapy (*n* = 250)

Symptom	*n* (%)	Mean (SD)	Sequencing of severity
Fatigue	210 (84.0)	1.14 (0.74)	2
Nocturia	208 (83.2)	1.44 (0.90)	1
Memory loss	186 (74.4)	0.93 (0.70)	5
Disturbed sleep	178 (71.2)	1.00 (0.78)	3
Dry mouth	170 (68.0)	0.87 (0.73)	6
Hot flushes	169 (67.6)	0.95 (0.82)	4
Restlessness	165 (66.0)	0.84 (0.78)	8
Lumbosacral soreness	163 (65.2)	0.86 (0.78)	7
Shortness of breath	159 (63.6)	0.82 (0.75)	9
Susceptible sigh	151 (60.4)	0.74 (0.73)	11
Lower abdominal pain	144 (57.6)	0.66 (0.65)	14
Numbness	140 (56.0)	0.72 (0.76)	12
Distress	139 (55.6)	0.72 (0.76)	13
Pain	137 (54.8)	0.64 (0.65)	15
Poor appetite	135 (54.0)	0.75 (0.83)	10
Dizziness	120 (48.0)	0.57 (0.66)	20
Abdominal distension	120 (48.0)	0.55 (0.66)	22
Spontaneous perspiration	119 (47.6)	0.60 (0.76)	18
Bitter taste	115 (46.0)	0.63 (0.83)	16
Sadness	112 (44.8)	0.54 (0.69)	24
Frequent micturition	110 (44.0)	0.52 (0.66)	26
Chills	109 (43.6)	0.61 (0.80)	17
Night sweats	109 (43.6)	0.58 (0.77)	19
Chest tightness	109 (43.6)	0.54 (0.70)	23
Urinary urgency	105 (42.0)	0.55 (0.76)	21
Difficult defecation	90 (36.0)	0.51 (0.78)	27
Tinnitus	84 (33.6)	0.39 (0.60)	30
Emaciation	83 (33.3)	0.53 (0.85)	25
Tenesmus	82 (32.8)	0.43 (0.71)	28
Loose stool	75 (30.0)	0.41 (0.73)	29
Dry stool	70 (28.0)	0.33 (0.58)	33
Urinary pain	69 (27.6)	0.31 (0.56)	34
Abnormal leucorrhea	68 (27.2)	0.38 (0.70)	31
Nausea	67 (26.8)	0.35 (0.65)	32
Edema	46 (18.4)	0.25 (0.56)	35
Drowsiness	35 (14.0)	0.14 (0.36)	36
Vomiting	23 (9.2)	0.11 (0.37)	37
Vaginal bleeding	18 (7.2)	0.10 (0.40)	38
Bloody purulent stool	13 (5.2)	0.06 (0.28)	39
Irregular menstruation	5 (2.0)	0.04 (0.27)	40

*SD* standard deviation

#### Symptom clusters

A total of 30 symptoms were included in this part, and 10 symptoms with a symptom prevalence < 30% were removed, which were dry stool, urinary pain, irregular menstruation, bloody purulent stool, vaginal bleeding, vomiting, drowsiness, edema, abnormal leucorrhea, and nausea. KMO test and Bartlett’s ball test were as follows: KMO = 0.849, approximate chi square = 2477.769, *P* < 0.001, indicating suitability for factor analysis. Nine symptom clusters were identified in this study by exploratory factor analysis. Symptom clusters were named according to their high loadings in the cluster or shared characteristics of multiple symptoms.

Factor 1 was named as psycho–emotion-related symptom cluster (variance contribution 10.812%, eigenvalue 3.244), including four symptoms: feelings of sadness, distress, restlessness, and susceptible sigh. Factor 2 was named as pain–disturbed sleep-related symptom cluster (variance contribution 8.444%, eigenvalue 2.533), including five symptoms: pain, lumbosacral soreness, lower abdominal pain, disturbed sleep, and fatigue. Factor 3 was named as menopausal symptom cluster (variance contribution 7.695%, eigenvalue 2.308), including three symptoms: spontaneous perspiration, night sweats, and hot flushes. Factor 4 was named as tinnitus–dizziness-related symptom cluster (variance contribution 7.374%, eigenvalue 2.212), including five symptoms: tinnitus, dizziness, chest tightness, abdominal distension, and chills. Factor 5 was named as urinary-related symptom cluster (variance contribution 6.700%, eigenvalue 2.010), including three symptoms: frequent micturition, urinary urgency, and nocturia. Factor 6 was named as dry mouth–bitter taste-related symptom cluster (variance contribution 6.585%, eigenvalue 1.976), including three symptoms: dry mouth, bitter taste, and shortness of breath. Factor 7 was named as intestinal-related symptom cluster (variance contribution 5.797%, eigenvalue 1.739), including three symptoms: tenesmus, loose stool, and difficult defecation. Factor 8 was named as memory loss–numbness-related symptom cluster (variance contribution 4.977%, eigenvalue 1.493), including two symptoms: memory loss and numbness. Factor 9 was named as emaciation-related symptom cluster (4.423% contribution of variance, eigenvalue 1.327), including two symptoms: emaciation and poor appetite (Tables 7 and 8).

**Table 7:** Exploratory factor analysis using symptom severity (*n* = 250)

Symptom	Factor 1	Factor 2	Factor 3	Factor 4	Factor 5	Factor 6	Factor 7	Factor 8	Factor 9
Sadness	0.841								
Distress	0.832								
Restlessness	0.771								
Susceptible sigh	0.700								
Pain		0.738							
Lumbosacral soreness		0.663							
Lower abdominal pain		0.544							
Disturbed sleep		0.530							
Fatigue		0.462							
Spontaneous perspiration			0.812						
Night sweats			0.802						
Hot flushes			0.792						
Tinnitus				0.641					
Dizziness				0.589					
Chest tightness				0.578					
Abdominal distension				0.413					
Chills				0.406					
Frequent micturition					0.793				
Urinary urgency					0.739				
Nocturia					0.736				
Dry mouth						0.719			
Bitter taste						0.691			
Shortness of breath				0.422		0.469*			
Tenesmus							0.767		
Loose stool							0.609		
Difficult defecation							0.528		
Memory loss								0.710	
Numbness								0.546	
Emaciation									0.831
Poor appetite						0.438			0.462*
Variance contribution ratio	10.812	8.444	7.695	7.374	6.700	6.585	5.797	4.977	4.423
Cumulative variance contribution	10.812	19.256	26.951	34.325	41.025	47.610	53.407	58.384	62.808
Characteristic value	3.244	2.533	2.308	2.212	2.010	1.976	1.739	1.493	1.327

“*” in the table indicates that the symptom load was greatest on this factor

**Table 8:** Symptom cluster severity (*n* = 250)

Factor	Symptom cluster	Mean (SD)
1	Pain–disturbed sleep-related symptom cluster	0.861 (0.498)
2	Urinary-related symptom cluster	0.836 (0.608)
3	Memory loss–numbness-related symptom cluster	0.826 (0.564)
4	Dry mouth–bitter taste-related symptom cluster	0.773 (0.580)
5	Menopausal symptom cluster	0.712 (0.650)
6	Psycho–emotion-related symptom cluster	0.711 (0.623)
7	Emaciation-related symptom cluster	0.640 (0.721)
8	Tinnitus–dizziness-related symptom cluster	0.530 (0.464)
9	Intestinal-related symptom cluster	0.449 (0.535)

*SD* standard deviation

Among the above nine symptom clusters, the three most severe symptom clusters were pain–disturbed sleep-related symptom cluster (0.861 ± 0.498), urinary-related symptom cluster (0.836 ± 0.608), and memory loss–numbness-related symptom cluster (0.826 ± 0.564) (Table 8).

## Discussion

Cervical cancer patients experience multisystem and multiorgan adverse effects after receiving radiotherapy and chemotherapy, which seriously affect patients’ mental health and quality of life. Symptom clusters as an evaluation tool for symptom management are able to extract a wide range of symptoms into clusters composed of 2 and more symptoms by using statistical methods [6]. Symptoms within clusters are thought to share common mechanisms [8]. This may provide support for further symptom cluster-based intervention studies. In clinical practice, it has been found that the scales used in the previous research on gynecological tumor symptom clusters, including cervical cancer, cannot fully reflect the symptoms experienced by patients, such as the MDASI scale and the MSAS scale [16, 17]. This may leave symptom clusters lacking in completeness. This study is the first to comprehensively investigate the symptom burden of cervical cancer patients within 6 months after radiotherapy and chemotherapy, preliminarily formed a clinical symptom questionnaire, and conducted a symptom cluster study.

### Symptom incidence and severity analysis

Fatigue has the highest incidence rate among the 40 symptoms, and its severity ranks second. This may be related to two reasons. One is related to the inflammatory process caused by cervical cancer itself and its treatment, and cancer fatigue may last for months or years after the end of treatment [18]; the other is related to factors such as the patient’s age, psychological state, and social pressure. In addition to fatigue, symptoms with an incidence > 50% and a severity in the top 10 were nocturia, disturbed sleep, hot flushes, memory loss, dry mouth, lumbosacral soreness, restlessness, shortness of breath, and poor appetite. These symptoms involve multi system, indicating the complexity of symptoms after chemoradiotherapy in cervical cancer patients. How to comprehensively intervene with complex symptoms without increasing the burden of patient treatment is one of the key issues to be addressed by clinical healthcare workers at present.

### Psycho–emotion-related symptom cluster

The psycho–emotion-related symptom cluster consists of sadness, distress, restlessness, and susceptible sigh. There have been cervical cancer symptom cluster studies identifying this symptom cluster [14]. Psycho–emotion-related symptom cluster results from multifaceted factors, such as illness distress, financial burden, communication disorders, and feelings of fear for the future. This symptom cluster has also been linked to the impact of inflammation induced by cancer and their associated treatments on behavior. Inflammatory cytokines can be involved in the development and progression of depression through interactions with various pathways, including monoamine metabolism, neuroendocrine function, synaptic plasticity, and neurocircuits relevant to mood regulation [19]. A study of symptom clusters in breast cancer survivors found that psychological and fatigue symptom clusters could be effectively improved by mindfulness-based stress reduction therapy [20].

### Pain–disturbed sleep-related symptom cluster

The pain insomnia-related symptom cluster, which consists of pain, lumbosacral soreness, lower abdominal pain, disturbed sleep, and fatigue, is the most experienced by patients. This symptom cluster has the highest severity. Pain, disturbed sleep, and fatigue often occur as a cluster of symptoms and are also common symptoms in cancer patients. A study of 1562 patients with advanced cancer identified four symptom clusters, including tense-worry-irritable-depressed (emotional cluster), fatigue-pain, nausea-vomiting, and concentration-memory (cognitive cluster), of which the fatigue-pain was a stronger predictor of overall health than the other clusters [21]. A potential common mechanism underlying pain, disturbed sleep, and fatigue may be a set of “sickness behaviors” triggered by inflammation and include cognitive dysfunction and affective symptoms that share a common cytokine-based neuroimmune mechanism [22, 23]. A study stated that the symptom cluster of insomnia patients with cancer can consist of insomnia, fatigue, anxiety, pain, depressed mood, and cognitive disorders, and the common biological mechanism may be related to brain inflammation by proinflammatory cytokine production, and proposed a homeopathic protocol for Belladonna 15c, Phosphorus 15c, Cerebral cortex 4c, and Nerves 4c to prevent chemotherapy-induced brain symptoms [12].

### Menopausal symptom cluster

The menopausal symptom cluster consists of spontaneous perspiration, night sweats, and hot flushes. The mean age of the patients in this study was 51.16 ± 10.98 years, which is at the stage of changes in hormone levels in the body around menopause. Hot flashes and sweating seem to be specific symptoms of perimenopausal onset [24]. In addition, cancer-related treatments such as surgery, radiotherapy, and chemotherapy can cause ovarian failure and further affect hormone levels, which can lead to autonomic dysfunction and cause symptoms such as spontaneous perspiration, night sweats, and hot flushes [25]. Previous studies have identified this symptom cluster. A longitudinal study of 115 ovarian cancer patients identified menopausal symptom cluster (lack of energy, dry mouth, difficulty sleeping, sweats, lack of appetite, and feeling irritated) and found this cluster remained consistent before and over the course of treatment [26]. Another study of 232 gynecological cancer patients undergoing chemotherapy identified hormonal, respiratory, psychological, and weight change symptom clusters in all dimensions and time points [27].

### Tinnitus–dizziness-related symptom cluster, dry mouth–bitter taste-related symptom cluster, memory loss–numbness-related symptom cluster, and emaciation-related symptom cluster

These four symptom clusters may be related to the adverse reactions caused by chemotherapy. Tinnitus–dizziness-related symptom cluster and memory loss–numbness-related symptom cluster are related to the neurotoxicity of chemotherapy. Dry mouth–bitter taste-related symptom cluster and emaciation-related symptom cluster are related to the gastrointestinal toxicity of chemotherapy. Neurotoxicity of chemotherapy, which causes damage to dorsal root ganglion neurons or their axons, leads to acral pain, sensory loss, and sometimes sensory ataxia, while motor, autonomic, and cranial nerve symptoms may also occur [28]. Chemotherapy-induced damage to the gastrointestinal mucosa, the release of inflammatory mediators and neurotransmitters, and altered sensation may lead to the development of one or even more gastrointestinal symptoms in patients [29]. However, this still cannot explain why chest tightness, abdominal distension, and chills appear in the tinnitus–dizziness-related symptom cluster and why shortness of breath occurs in the dry mouth–bitter taste-related symptom cluster, and further research is needed.

### Urinary-related symptom cluster and intestinal-related symptom cluster

These two symptom clusters may be related to adverse reactions caused by radiotherapy. Urinary-related symptom cluster consists of frequent micturition, urinary urgency, and nocturia, which may be related to radiation cystitis. Since the bladder mucosa is damaged by radiation, there is congestion, mucosal edema, and inflammation, which are self-limiting; the symptoms at presentation usually last up to 3 months [30]. The intestinal-related symptom cluster consisted of tenesmus, loose stools, and difficult defecation, which may be related to radiation enteritis after radiotherapy. Radiation enteritis can present as an acute or chronic syndrome, with the acute form developing within hours to days of radiation exposure and the chronic form developing within 2 months or 30 years of exposure [31]. Clinical findings show that these two groups of symptoms are delayed and persistent, especially common in middle-aged and elderly people, and the pathogenic mechanisms are related to inflammatory damage.

### Research methods for symptom clusters

As the study of symptom clusters expands and deepens, its research methods also continue to be refined. The research methods of symptom cluster research are divided into a priori identification and empirical identification [3]. A priori identification refers to methods for predetermining the composition of symptom clusters based on patient experiences, clinical observations of symptom co-occurrence, or research hypotheses on symptom relationships. But this method could not solve the problem of completeness of symptom cluster; meanwhile, part of symptoms would be missed by preset [3]. The empirical identification refers to the use of statistical methods to extract symptoms from the patient’s symptom library and identify symptom clusters, wherein the patient’s symptom library consists of one or more symptom evaluation scales. This method is currently a widely used clinical symptom cluster identification method, mainly including principal components analysis (PCA), common factor analysis (CFA), and hierarchical cluster analysis (HCA) [32, 33]. In the further identification of subgroups of symptom clusters, the researchers surveyed the symptoms in the identified symptom clusters using the symptom rating scale, and applied cluster analysis [34], latent class analysis (LCA) [35], latent profile analysis (LPCA) was performed for subgroup identification analysis [10].

The use of empirical identification for symptom group research will be disturbed by two aspects: one is the symptom assessment scale, and the other is the analysis method. In other words, the number of symptom clusters and the number of symptoms within a symptom cluster can be affected by the number of symptom assessment scale entries and the method of analysis. In this study, through a comprehensive investigation of the symptom burden of patients, 40 symptoms were screened out, 30 symptoms were included for exploratory factor analysis, and 9 symptom groups were obtained, which involved multiple systems. If the research is limited to the selection of expected scales, a symptom may be missed in a certain symptom cluster, which will lead to a lack of specificity in the symptom cluster.

## Limitation

There are several limitations worth considering. This study comprehensively investigated the symptom burden of patients, but the symptom survey tool used has not been analyzed for reliability and validity. Follow-up research can formulate a qualified PRO scale on this basis to meet the needs of clinical research. We only conducted a cross-sectional survey on patients within 6 months after radiotherapy and chemotherapy for cervical cancer. If we can conduct surveys at multiple time points, the research results will be richer.

## Conclusion

Symptoms in patients with cervical cancer within 6 months after radiotherapy and chemotherapy involve multiple systems and organs. We were able to identify nine symptom clusters based on the incidence and severity of symptoms reported in the patient symptom burden survey. Through preliminary exploration, combined with previous mechanism research and clinical research, the potential biological mechanism of each symptom group can be found, but the specific and clear mechanism still needs further research. Through the survey of symptom burden, this study found that the number of symptom clusters and the number of symptoms within a symptom cluster were closely related to the symptom assessment scale selected in the study. Therefore, the study of symptom groups urgently needs a more targeted symptom assessment scale applicable to different cancer and different cancer stages to determine symptom groups, so that symptom management decisions based on this can be representative.

Therefore, the study of symptom clusters urgently needs a more targeted symptom assessment scale applicable to different cancer sites, different cancer stages, and different cancer treatment stages to determine symptom clusters, based on which symptom management decisions are more representative.

## Data Availability

Not applicable.
